# Cu-Enhanced Bottlebrush Composite Polymer Electrolytes for Superior Mechanical and Electrochemical Performance

**DOI:** 10.1021/acsaem.5c02545

**Published:** 2025-10-28

**Authors:** So Young An, Brian Hu, Young-Geun Lee, Yuqi Zhao, Ting-Chih Lin, Jay F. Whitacre, Krzysztof Matyjaszewski

**Affiliations:** † Department of Chemistry, 6612 Carnegie Mellon University, 4400 Fifth Avenue, Pittsburgh, Pennsylvania 15213, United States; ‡ Department of Materials Science and Engineering, Carnegie Mellon University, 5000 Forbes Avenue, Pittsburgh, Pennsylvania 15213, United States; § Scott Institute for Energy Innovation, Carnegie Mellon University, 5000 Forbes Avenue, Pittsburgh, Pennsylvania 15213, United States

**Keywords:** polymer electrolyte, composite polymer electrolyte, battery materials, lithium metal batteries, bottlebrush polymer electrolyte

## Abstract

The development of safe and high-performance electrolytes is essential to realize the full potential of lithium metal batteries (LMBs) for next-generation energy storage. In this study, we report the design and synthesis of composite polymer electrolytes (CPEs) based on polyoxanorbornene bottlebrush polymers (BPs) with poly­(ethylene oxide) (PEO) side chains. These unique bottlebrush architectures, synthesized via ring-opening metathesis polymerization, enable precise control over mechanical properties while maintaining a high ionic conductivity. The incorporation of copper bis­(trifluoromethanesulfonyl)­imide (Cu­(TFSI)_2_) into the polymer matrix enhances ionic conductivity by disrupting PEO crystallinity and modifying the local lithium coordination environment. Electrochemical impedance spectroscopy revealed that the optimized CPE with 2 wt % Cu­(TFSI)_2_ exhibited a 3-fold increase in ionic conductivity compared to BPs without Cu salt incorporation. Symmetric Li|Li cells demonstrated stable cycling with low overpotential for over 500 h, highlighting the electrolyte’s excellent lithium metal compatibility and dendrite suppression capabilities. Full-cell tests with LiFePO_4_ (LFP) and perylenetetracarboxylic dianhydride (PTCDA) cathodes further confirmed the electrolyte’s versatility, delivering high capacities, superior rate performance, and extended cycle life compared to conventional polymer electrolytes. This work demonstrates that Cu-modified bottlebrush polymer electrolytes are a promising platform for enabling high-performance, solid-state LMBs, with broad applicability to both inorganic and organic cathodes.

## Graphical abstract

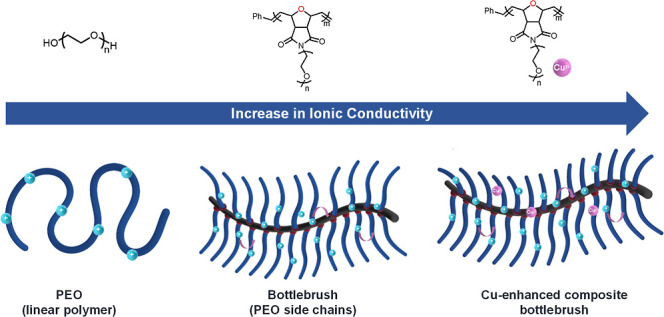

## Introduction

Over the last ten years, the production of lithium-ion batteries (LIBs) has skyrocketed. This surge is expected to continue at a rapid pace, primarily due to the increasing adoption of electric vehicles and growing energy storage demand.^1,2^ As industries push for next-generation energy storage solutions, there is a strong need for batteries that offer higher energy density, enhanced safety, and lower costs. Despite their widespread use, conventional LIBs face significant limitations, particularly due to their limited energy density (∼250 Wh/kg).^3,4^ This limitation restricts their effectiveness in high-energy-demand applications, where a greater performance is essential.

To enhance the specific energy of rechargeable batteries, researchers have explored various high-energy electrode materials, including lithium, sodium, and silicon anodes.^5–11^ Among these, lithium metal anodes have proven to be one of the most promising solutions due to their exceptionally high theoretical capacity (∼3860 mA h/g), low electrochemical potential (−3.04 V vs SHE), and ability to significantly enhance the energy density of lithium-based batteries.^12,13^ Unlike conventional graphite anodes, which are limited to ∼372 mA h/g, lithium metal anodes can store a substantially higher charge, making them ideal candidates for next-generation energy storage systems such as lithium–sulfur (Li–S) and lithium–air (Li–O_2_) batteries.^14–17^ However, despite their advantages, the widespread adoption of lithium metal anodes faces major challenges, primarily due to their incompatibility with conventional nonaqueous liquid electrolytes.^18–21^ These electrolytes, which are highly volatile and flammable, pose significant safety risks, especially due to the uncontrolled growth of lithium dendrites. The formation of these dendrites during charge and discharge cycles leads to electrical instability, increasing the likelihood of short circuits, fires, and even explosions. To safely integrate high-energy metal anodes, it is crucial to develop alternative electrolyte systems that enable stable and efficient battery operation.^22^

Solid-state electrolytes have emerged as a promising solution, offering superior thermal and chemical stability while effectively mitigating dendrite formation in lithium.^23^ Among the various candidates, solid polymer electrolytes (SPE) stand out for their potential to enable high-energy-density batteries.^24–34^ Their key advantages—low cost, high processability, and seamless integration into existing lithium metal battery architectures—make them a more adaptable choice compared to ceramic solid electrolytes. For instance, PEO-based solid polymer electrolytes (SPEs) have been extensively investigated due to PEO’s repeating ethylene oxide units, which facilitate salt dissociation and ionic transport through ion hopping or segmental motion of polymer chains. However, the crystallinity of PEO must be carefully managed, as it significantly restricts the ionic conductivity of these materials at ambient conditions (≈10^–8^ to 10^–5^ S cm^–1^ at room temperature).^24,26,35,36^

Recently, the main focus for SPE has been to increase the amorphous phase of the polymers to boost the ionic conductivity since the ion conduction in the majority polymer electrolyte occurs in the amorphous region. To improve polymer segmental dynamics and create additional Li^+^ transport channels, researchers have investigated higher ordered structured polymer architectures, including block copolymers, cross-linked or branched networks, and bottlebrush polymers (BPs). Among these, non-linear designs such as bottlebrush-like structures, synthesized via atom transfer radical polymerization (ATRP) and ring-opening metathesis polymerization (ROMP), have exhibited enhancements in ionic conductivity—up to 3 orders of magnitude higher than linear PEO.^37–47^ Recently, a new class of solid polymer electrolytes (SPEs) was introduced, featuring polyoxanorbornene-based BPs with PEO side chains synthesized through ROMP.^48^ This dual-conductive system, utilizing both the PEO side chains and the polyoxanorbornene backbone, enables a high ionic conductivity at room temperature along with an outstanding electrochemical performance. While these advancements highlight the critical role of molecular engineering in developing stable and highly conductive polymer electrolytes for lithium metal batteries (LMBs), current design approaches still face challenges related to mechanical strength, and their ionic conductivity remains constrained by the length of conductive polar chain repeat units.

Composite polymer electrolytes (CPEs) offer significant advantages over conventional SPEs by combining the flexibility and processability of polymers with the enhanced ionic conductivity and mechanical reinforcement provided by inorganic fillers.^49–51^ Common fillers such as Li_7_La_3_Zr_2_O_12_ (LLZO), Li_1.3_Al_0.3_Ti_1.7_ (PO_4_)_3_ (LATP), SiO_2_, Al_2_O_3_, and TiO_2_ improve ion transport, mechanical stability, and dendrite suppression. However, these fillers also have limitations.^52–56^ The high cost of ceramic fillers, such as LLZO and LATP, can be a barrier to large-scale applications. Additionally, achieving high conductivity while maintaining processability requires an optimal balance of the filler content. A minimum filler loading of approximately 5–10 wt % is generally necessary to significantly enhance ionic conductivity and mechanical stability.^57,58^ Recently, a PEO-based composite electrolyte with CuF_2_ demonstrated a copper-ion coordination effect with both PEO and lithium salt, leading to enhanced Li^+^ conductivity and an improved transference number at 30 °C.^59^ While this approach successfully formed hybrid networks through molecular engineering, the reliance on linear PEO architecture as conductive sites highlights the limitations of conventional polymer backbones in achieving both high mechanical strength and ionic conductivity.

Unlike conventional PEO-based SPE, CPEs featuring BPs—the focus of this study—demonstrates intrinsically superior mechanical stability and enhanced ionic conductivity, making them a compelling alternative for next-generation energy storage. In this work, we employed CPEs utilizing polyoxanorbornene bottlebrush architectures that enhance mechanical robustness without compromising ion transport. The unique bottlebrush structure enables precise control over mechanical properties by adjusting the molecular weight of the polymer’s side chains and backbone, achieving an optimal balance between rigidity and flexibility. Additionally, the integration of Cu ions within the CPE matrix disrupts crystallization and modifies the ionic coordination environment (O–Li interactions), facilitating faster Li-ion transport (Figure 1). This tailored material design results in CPEs with exceptional mechanical durability and high electrochemical performance, making them promising candidates for next-generation energy storage technologies. Notably, we further demonstrate the versatility of this CPE platform by applying it to both lithium metal anode and organic cathode systems. Perylenetetracarboxylic dianhydride (PTCDA), a redox-active organic material, is capable of reversible two-electron, two-lithium-ion reaction but suffer from dissolution and stability issues in conventional liquid electrolyte.^60,61^ The CPE electrolyte mitigates this solubility challenge through physical confinement, enabling stable cycling and extending the utility of organic cathodes. These findings highlight the broad applicability of bottlebrush-based CPEs as a robust and adaptable platform for a wide range of lithium battery chemistries.

**Figure 1:**
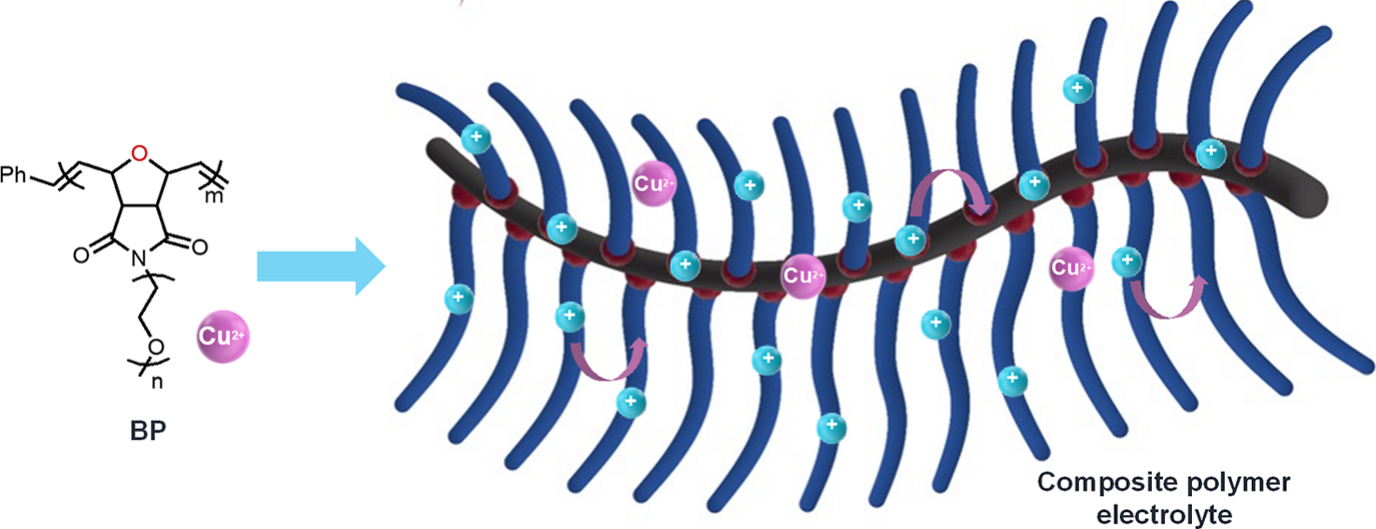
Preparation of CPE based on polyoxanorbornene BP.

## Results and Discussion

### Synthesis and Characterization of Bottlebrush Polymers

We developed a series of polymers incorporating poly­(ethylene oxide) (PEO) repeat units attached to a polyoxanorbornene backbone. The synthesis process followed established methods reported in previous studies.^48^ Macromonomers containing monomethoxy-terminated poly­(ethylene oxide) (*M*_n_,_avg_ = 2147 g/mol) were prepared by using a Mitsunobu-type coupling reaction. These macromonomers were subsequently polymerized through ROMP using a third-generation Grubbs catalyst (G3). Polymerization was carried out at varying monomer-to-catalyst ratios to control the degree of polymerization (DP), with monomer concentrations ranging from 0.14 to 0.04 M in dichloromethane. The resulting BPs were designated as BP-1, BP-2, and BP-3, with detailed polymerization conditions summarized in Table 1. A complete macromonomer consumption was obtained for all BPs by ROMP, and macromonomer conversion was assessed by tracking the disappearance of vinyl proton peaks at 6.50 ppm and the emergence of polymer proton signals between 6.12 and 5.71 ppm in ^1^H NMR (Figures S2 and S3). A study by Ji et al. demonstrated that incorporating grafted PEO side chains can significantly improve ionic conductivity.^46^ In particular, PEO side chains with a number-average molecular weight (*M*_n_) ranging from 300 to 950 exhibited up to a two-order-of-magnitude increase in room-temperature conductivity. Despite this enhancement, their molecular weight remains below the PEO’s entanglement threshold in order to maintain amorphous PEO regions for good ionic conductivity. In contrast, the BPs in this work feature longer side chains (*M*_n_ ∼ 2147 g/mol) with DPsc (DP of side chains = 45) incorporated into the bottlebrush backbone. Using a bottlebrush synthesis approach, we designed high-molecular-mass BPs with side chains exceeding the entanglement threshold to enhance mechanical stability. All BPs exhibited good thermal stability after polymerization confirmed by thermal gravimetric analysis (TGA). The excellent thermal resilience of the SPEs (>200 °C) is also crucial, given the increasing safety concerns surrounding LIBs and the demanding operating conditions of LMBs (Figure S4).

**Table 1:** Results of ROMP of Macromonomer to Prepare a Series of BPs^a^

entry	[G3]/[M]	*M*_*n*,theo_ (kg/mol)	conv (%)	*M*_*n*_ ^b^_,GPC_ (kg/mol)	*D̵*
BP-1	1:100	215	≈100	159	1.11
BP-2	1:150	322	≈100	221	1.30
BP-3	1:250	229	≈100	267	1.42

a Reaction conditions: monomer conversion was determined by using ^1^H NMR spectroscopy. All measurements were analyzed using GPC (dimethylformamide as eluent) calibrated to poly­(methyl methacrylate) standards.

b Absolute molecular weight (*M*_n_, _MALS_).

### Preparation of Composite Polymer Electrolytes

CBP–Li-Cu (composite BP electrolytes) were formulated using these BPs with the addition of Cu­(TFSI)_2_ through a casting method, where the EO/Li^+^ ratios and content of Cu­(TFSI)_2_ were carefully tuned. The ethylene oxide/lithium ion (EO/Li^+^) ratio significantly influences the ionic conductivity, mechanical properties, and processability of polymer electrolytes. Compared with the other ratios, a 10/1 = EO/Li^+^ ratio typically exhibits the highest ionic conductivity due to the increased concentration of charge carriers, facilitating enhanced ion transport (Figure 2D). Additionally, at this ratio, the electrolyte maintains sufficient amorphous content, which is critical for effective lithium-ion mobility, while still being easier to process than higher-ratio formulations. Note that increasing the salt content beyond this level can lead to excessive ion pairing and reduced mechanical integrity. Previous studies indicated that Cu^2+^ ions altered the local Li–O coordination environment by forming Cu–O bonds, thereby reducing the number of oxygen atoms from EO units and other heteroatoms available to interact with Li^+^.^62^ A similar phenomenon was observed in our CBP–Li-Cu, with FT-IR analysis (Figures S5 and S6) confirming the chemical interactions among EO units, LiTFSI, and Cu­(TFSI)_2_. FT-IR spectra of CBP-2 (EO/Li^+^ = 10/1) with and without 2 wt % Cu­(TFSI)_2_ show characteristic EO peaks, including C–H bending (∼1460 cm^–1^), CH_2_ stretching (∼2870 cm^–1^), and C–O–C stretching (∼1100 cm^–1^). Upon Cu­(TFSI)_2_ addition, a slight shift and intensity change in the 1127 and 1190 cm^–1^ region (−CF_3_ symmetric and asymmetric stretching) indicates altered ionic coordination, likely due to Cu^2+^ interaction with the TFSI^–^ anion. The C–O stretching region (∼946 cm^–1^) also exhibits a minor shift, suggesting interactions between Cu^2+^ and ether oxygens in the PEO chain. Next, we calculated ionic conductivity (eq S1 using electrochemical impedance spectroscopy (EIS, see the corresponding values in Table 1). At 25 °C, the CBP-2 electrolyte without Cu­(TFSI)_2_ incorporation exhibited a conductivity of 8.51 × 10^–5^ S cm^–1^, while CBP-2 demonstrated close to a 3-fold increase (2.48 × 10^–4^ S cm^–1^) with the incorporation of 2% Cu­(TFSI)_2_ (Figures 2, S7 and Table 2). In conventional ether-based polymer electrolytes, low Li-ion conductivity arises due to strong Li^+^-O interactions in the solvation structure, limiting ion mobility.^63^ The presence of Cu^2+^ ions disrupt these interactions, weakening Li^+^-O coordination and promoting faster ion transport, thereby improving overall conductivity.^59,64,65^ However, an excess of Cu^2+^ ions leads to undesirable competition for conductive sites, as these ions begin to occupy heteroatomic coordination sites, hindering the intrinsic Li^+^ transport pathways. This effect was evident in CBP-2, where adding Cu­(TFSI)_2_ beyond 2 wt % caused a gradual decline in ionic conductivity, ultimately falling below that of CBP-2 electrolytes without Cu­(TFSI)_2_ incorporation. The BPs exhibit a dual ion conduction mechanism, facilitated by heteroatoms present on both the backbone and side chains.^48^ Consequently, the DP of the backbone (DP_bb_) plays a significant role in determining the overall ionic conductivity. As a result, CBP-1 (DP_bb_ = 100) demonstrated lower ionic conductivity compared to that of the CBP-2 series (DP_bb_ = 150), regardless of the amount of Cu­(TFSI)_2_ incorporated. Interestingly, an inverse relationship between ionic conductivity and Cu content was observed for CBP-1, where conductivity declined with increasing Cu incorporation. This behavior arises because the polyoxanorbornene backbone participates in both Li-ion and Cu-ion coordination, and shorter bottlebrush structures lead to earlier saturation of Cu–O bonds within conductive sites, producing trends distinct from those seen in CBP-2. On the other hand, CBP-3 (DP_bb_ = 250) exhibited a similar trend in ionic conductivity, reaching its peak conductivity at 2 wt % Cu­(TFSI)_2_ incorporation. However, CBP-3 at 2 wt % Cu­(TFSI)_2_ displayed significantly lower conductivity than CBP-2, likely due to the increased rigidity of the polymer backbone at higher DP_bb_ values (DP_bb_ = 250). Note that the polyoxanorbornene backbone with high DP_bb_ becomes increasingly rigid due to the inherent conformational constraint imposed by the double bond within the norbornene structure, which limits rotational freedom and promotes a stiff chain configuration. Similar observations regarding polyoxanodrbornene-derived polymer backbones and their rigidity at higher DP have been discussed in prior studies.^48^ Beyond a certain threshold, this structural rigidity hinders ion transport along the oxanorbornene backbone, limiting the overall conductivity.

**Figure 2:**
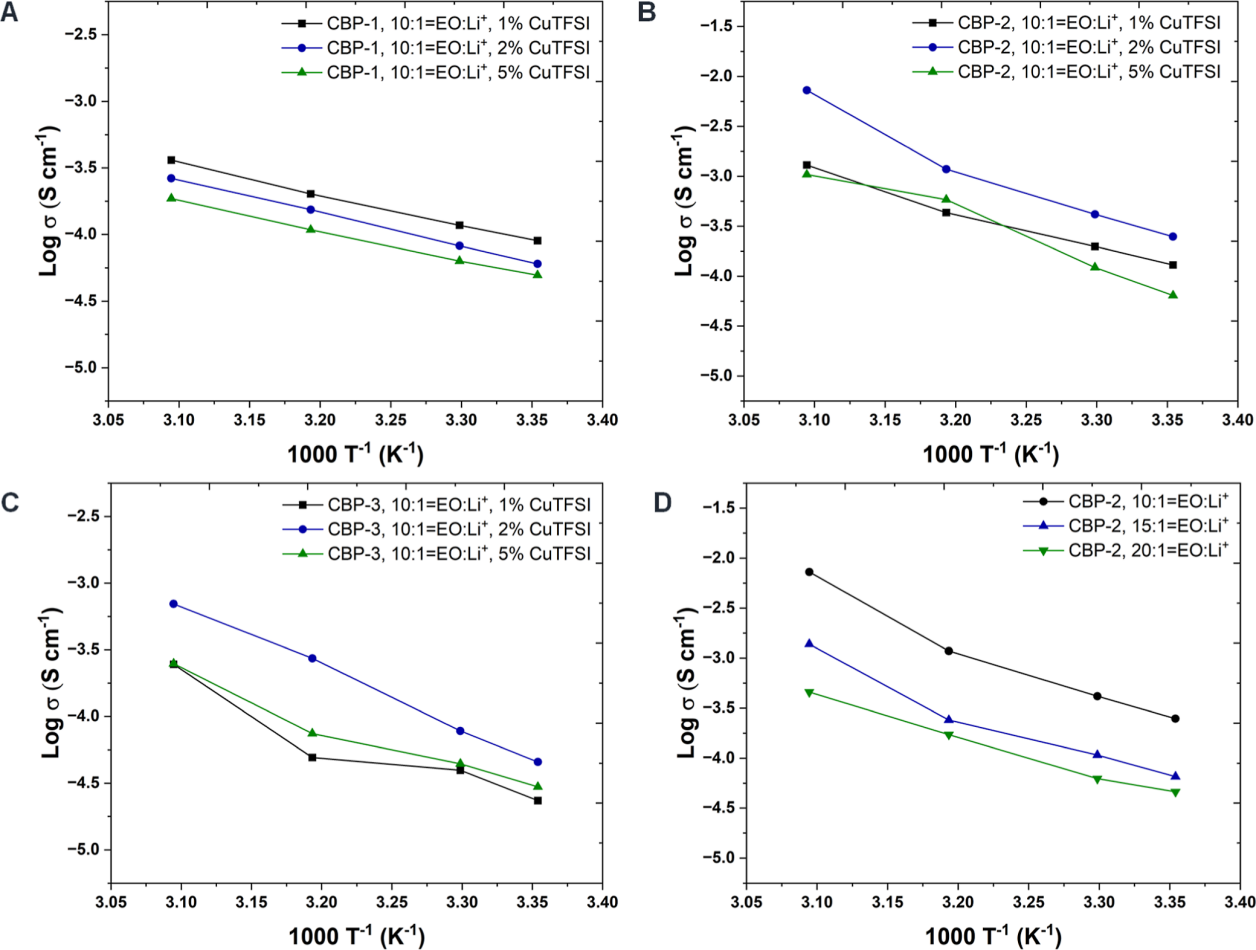
Ionic conductivity of CPEs with various weight % of Cu­(TFSI)_2_ for (A) CBP-1, (B) CBP-2, (C) CBP-3, and (D) CBP-2 with set 2 wt % Cu­(TFSI)_2_ and different ratio of EO: Li^+^.

**Table 2:** Summary of Ionic Conductivity of CBPs

entry	Cu(TFSI)_2_ (%)	DP	[EO]/[Li+]	σ (S cm-1) at RT	σ (S cm-1) at 40 °C
CBP-1	1	100	10:1	8.98 × 10^–5^	2.02 × 10^–4^
CBP-1	2	100	10:1	6.02 × 10^–5^	1.53 × 10^–4^
CBP-1	5	100	10:1	4.95 × 10^–5^	1.08 × 10^–4^
CBP-2	1	150	10:1	1.30 × 10^–4^	4.32 × 10^–4^
CBP-2	2	150	10:1	2.48 × 10^–4^	1.18 × 10^–3^
CBP-2	5	150	10:1	6.40 × 10^–5^	5.82 × 10^–4^
CBP-2	2	150	15:1	6.54 × 10^–5^	2.41 × 10^–4^
CBP-2	2	150	20:1	4.61 × 10^–5^	1.72 × 10^–4^
CBP-2	0	150	10:1	8.51 × 10^–5^	3.17 × 10^–4^
CBP-3	1	250	10:1	2.34 × 10^–5^	4.92 × 10^–5^
CBP-3	2	250	10:1	4.56 × 10^–5^	2.72 × 10^–4^
CBP-3	5	250	10:1	2.97 × 10^–5^	7.45 × 10^–5^

A differential scanning calorimeter (DSC) was utilized to examine the phase transition behavior of the BP and CBP electrolytes (Figure S9). The DSC analysis revealed that the CBP-2 without Cu electrolyte exhibited a glass transition temperature (*T*_g_) at −42.1 °C, while the CBP-2 with 2 wt % Cu electrolyte showed a lower *T*_g_ at −49.6 °C, with no detectable melting temperature (*T*_m_). These findings suggest that the crystallinity of long PEO chains was effectively suppressed due to the strong interactions between Li^+^ ions and EO units. Additionally, the results imply that the mobility of EO chains and the polymer backbone in the bottlebrush structure increase in the presence of Cu­(TFSI)_2_, contributing to the disrupted crystallization in Cu-incorporated CBP-2 electrolytes. Previously, a slight increase in tensile stress accommodation was reported by a polymer electrolyte sample with *M*_n_ = 950 with a value of 2.6 MPa, which was only marginally higher than that of linear PEO electrolyte with *M*_n_ = 100,000.^47^ On the other hand, CBP-2 after 2 wt % of Cu­(TFSI)_2_, exhibited much higher values of both *G*′ and *G*″ (an order of magnitude higher than CBP-2 without Cu­(TFSI)_2_), indicating significantly enhanced mechanical strength and elasticity (Figure S10). This enhancement is attributed to the incorporation of Cu­(TFSI)_2_ and the resulting bottlebrush architecture, which impart increased network rigidity and energy dissipation capacity. In contrast, linear PEO displayed the lowest *G*′ and *G*″, reflecting inferior mechanical integrity prior to any structural modifications. These findings underscore the mechanical superiority of the Cu­(TFSI)_2_-modified bottlebrush electrolyte compared with the conventional linear PEO system. Based on this evaluation, CBP-2 containing 2 wt % Cu­(TFSI)_2_ was identified as the optimal formulation (hereafter referred to as CBP-2-Cu), as it exhibited the highest ionic conductivity among the synthesized polymers. Consequently, this formulation was selected for all subsequent electrochemical investigations. The corresponding baseline formulation without Cu­(TFSI)_2_ incorporation is termed the BP-2 electrolyte.

## Electrochemical Characterization of Composite Polymer Electrolytes

Symmetric Li|Li cells were prepared to assess the lithium metal stability of the CBP-2-Cu and BP-2 electrolytes (Figure 3). The cells underwent 1 h charge and discharge cycles at current densities of 0.1, 0.2, and 0.5 mA cm^–2^, before returning to 0.1 mA cm^–2^, all at room temperature. In these tests, Li-ion plating and stripping on the lithium electrodes simulated real charging and discharging operations that would be experienced at the negative electrode of a solid-state lithium metal battery. The cells containing the CBP-2-Cu electrolyte maintained a consistent, low overpotential plateau of 0.13 V at 0.1 mA cm^–2^, 0.15 V at 0.2 mA cm^–2^, and 0.28 V at 0.5 mA cm^–2^. When the current density was reduced back to 0.1 mA cm^–2^, the overpotential dropped to 0.12 V, though it gradually rose to 0.22 V after 450 h of cycling. In contrast, cells with the BP-2 electrolyte showed much higher overpotentials at 0.1 and 0.2 mA cm^–2^, experiencing multiple soft short circuits and eventually a significant high overpotential event at 0.5 mA cm^–2^. At 0.1 mA cm^–2^, the steady overpotential was ≈0.50 V for the baseline BP-2 electrolyte compared to ≈0.13 V for CBP-2-Cu, consistent with the 3-fold enhancement in conductivity observed by EIS. Furthermore, the incorporation of Cu^2+^ into the BP electrolyte leads to a slight extension of the electrochemical stability window according to the linear sweep voltammetry (LSV) results (Figure S11). This effect is likely due to the coordination of Cu^2+^ with ether oxygen sites, which reduces the number of polymer sites available for oxidative decomposition. Although the improvement is modest, such Cu^2+^–polymer interactions can help suppress early oxidative process.

**Figure 3:**
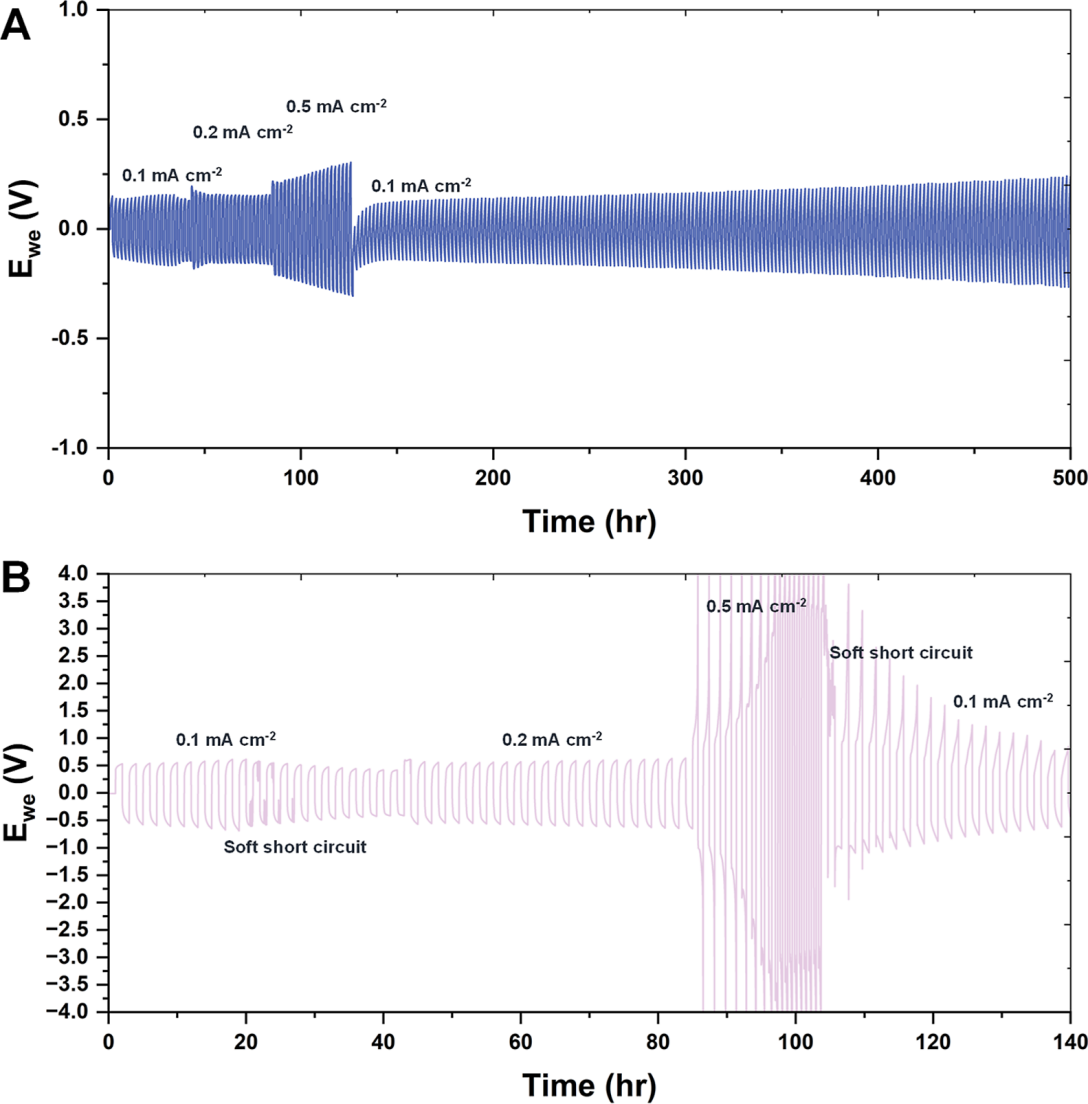
Voltage–time curve of the (A) Li|CBP-2-Cu|Li (B) Li|BP-2|Li.

To further understand the changes in Li electrode morphology during Li|Li symmetric cycling, SEM images of the Li anodes were analyzed after certain cycle numbers. Initially, the CBP-2-Cu system displayed a smooth, flat surface, which indicated excellent contact between the composite electrolyte and the Li metal. This uniform composite polymer layer remained intact for up to 50 and 100 h. However, under more extensive cycling (400 h of cycling), the polymer layer developed porosity and evolved into an additional organic/Li composite structure. Despite the interphase between the Li electrode and the polymer layer becoming more heterogeneous and porous over prolonged cycling, there were no observable dendrites or cracks. These post-mortem SEM observations imply that CBP-2-Cu can effectively suppress dendritic growth while maintaining strong interfacial compatibility with the Li metal anode (Figure 4).

**Figure 4:**
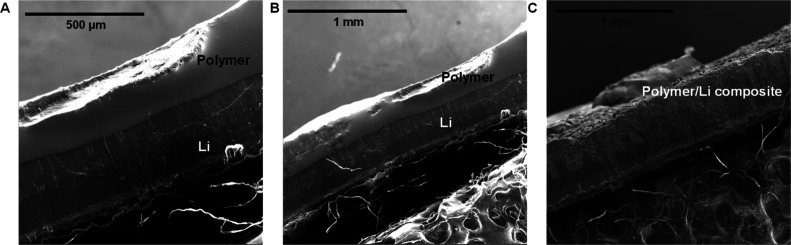
SEM images of cycled lithium anode from Li|CBP-2-Cu|Li (A) after 50 h and (B) after 100 h (C) after 400 h.

To demonstrate the competitiveness of Li metal batteries, we evaluated the CBP-2-Cu electrolyte in a full-cell configuration by using LFP as the cathode. Here, relatively low cathode mass loading was employed to evaluate electrolyte compatibility in the LMB system. Higher loading could be achieved by incorporating catholyte functionality to facilitate ion transport within the electrode or by adopting strategies such as hybrid polymer-ceramic composites or modifying the cathode lattice.^66–69^ We assessed the rate capability of the LFP cathode with our P1 electrolyte at current densities ranging from 0.1 to 2 C-rate, employing an asymmetric charge/discharge protocol to determine the feasibility of CBP-2-Cu in LMBs. As illustrated in Figure 5A, the discharge capacity of the Li|CBP-2-Cu|LFP cell gradually decreased with increasing current density, delivering 154.8, 148.2, 134.5, 119.2, and 98.58 mAh g^–1^ at 0.1, 0.2, 0.5, 1, and 2 C-rates, respectively. In contrast, the Li|BP-2|LFP cell, which lacks Cu^2+^ incorporation, showed significantly lower reversible capacities, delivering 100.5, 92.2, 77.9, 60.2, and 31.8 mAh g^–1^ at 0.1, 0.2, 0.5, 1, and 2 C-rates, respectively. The superior capacity retention of CBP-2-Cu becomes evident at higher current densities, where it is able to retain 63.6% of its initial capacity, while BP-2 exhibits a much lower recovery rate, with only 31.6% of its initial capacity restored. Upon returning the C-rate to 0.1C from a higher current rate, both CBP-2-Cu and BP-2 demonstrated a nearly complete restoration of their initial capacity after just one cycle.

**Figure 5:**
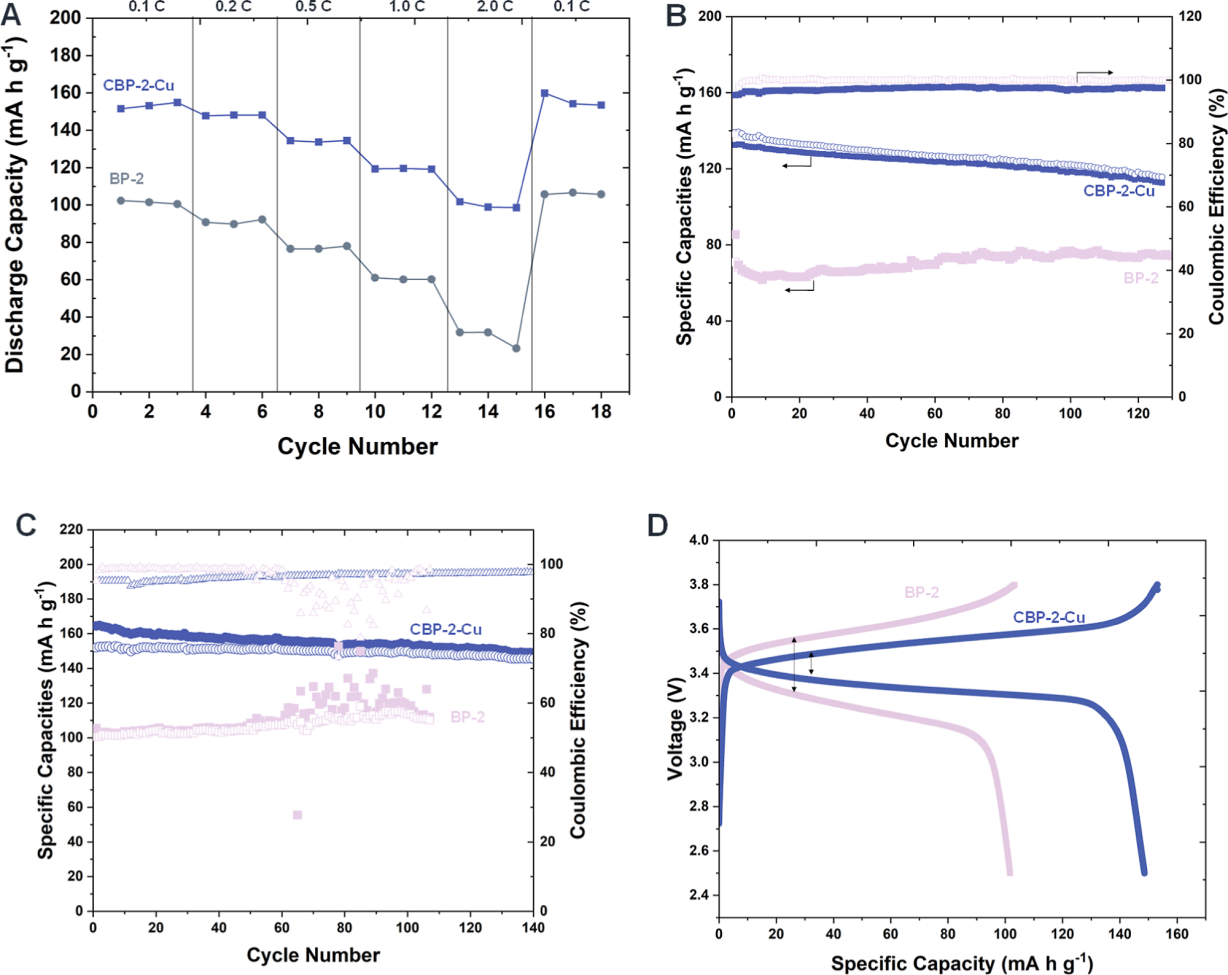
Electrochemical performance of CBP-2-Cu with the LFP cathode at room temperature. (A) Rate capability. (B) Cycling stability at 0.5C. (C) Cycling stability at 0.1C. (D) Charge/discharge profiles at 0.1C.

To assess the stability of Li|CBP-2-Cu|LFP cells, we performed cycling tests over 120 cycles within a voltage range of 3.8 to 2.5 V (Figure 5B). These tests involved asymmetric charge–discharge cycles at uniform current densities. Compared to their BP-2 counterparts (92.1 mAh g^–1^), CBP-2-Cu demonstrated higher specific capacities (132.5 mA h g^–1^ in the early stages of cycling) and retained 96.1% of their initial capacity over 120 cycles, maintaining a relatively high Coulombic efficiency (∼98%). Although CBP-2-Cu exhibited a slightly lower Coulombic efficiency compared with the ideal benchmark BP-2, this minor reduction can be attributed to side reactions involving redox-active Cu species or interfacial stability by Cu^2+^ incorporation. Nevertheless, the formulation demonstrated significantly improved specific capacities and stable cycle performance, highlighting the beneficial role of Cu^2+^ in enhancing the electrochemical activity despite the modest compromise in efficiency. The superior cycling stability and rate capability of CBP-2-Cu can be attributed to its BP architecture and the incorporation of Cu, which facilitates the formation of an effective CPE. The unique dual conduction pathway in CBP-2-Cu, along with the Cu–O interactions, enhances effective Li-ion binding/release and transport, thereby improving ionic conductivity. Additionally, the high molecular weight of CBP-2 provides enhanced mechanical strength, suppresses dendrite formation, and ensures robust electrode contact, ultimately supporting long-term stability.

Lithium deposition morphology is highly dependent on current density. At low current densities, prolonged deposition promotes mossy or dendrite growth, whereas more compact and uniform plating is often observed at moderate rates. In this study, we also examined performance at low C-rate cycling, where such morphology-related challenges are pronounced, to further access the compatibility of our CPE with lithium metal.^70–72^ We assessed its electrochemical performance under a rather slower discharge rate (0.1C), which was a more harsh condition as a comparison (Figure 5C). The Li|CBP-2-Cu|LFP cell exhibited stable capacities of 151.9 mAh g^–1^, retaining 95.8% of its initial capacity after 140 cycles, demonstrating a performance trend similar to that observed at 0.5C. In contrast, the Li|BP-2|LFP cell delivered significantly lower initial capacities (100.5 mAh g^–1^) and experienced a more pronounced decline in Coulombic efficiency at an early stage when cycled at 0.1C, ultimately leading to a hard short. The inferior cycling stability of BP-2 at low C-rates compared to CBP-2-Cu is likely attributed to its weaker compatibility with the lithium metal. At 0.1C, lithium deposition occurs over a prolonged period, increasing the likelihood of uneven ion aggregation, which promotes mossy or dendritic growth in the BP-2 electrolyte. In contrast, the incorporation of Cu^2+^ in CBP-2-Cu enhances its mechanical properties, effectively suppressing dendrite formation and maintaining stability under challenging cycling conditions for lithium metal anodes. The voltage profiles of the Li|CBP-2-Cu|LFP and Li|BP-2|LFP cells during 0.1C cycling, as shown in Figure 5D, demonstrates the electrochemical performance of both electrolytes. The Li|CBP-2-Cu|LFP cell exhibits a well-defined and stable charge–discharge plateau, indicative of efficient lithium-ion transport and minimal polarization. In contrast, the Li|BP-2|LFP cell shows an earlier voltage drop and a significantly lower discharge capacity, suggesting higher internal resistance and poorer interfacial stability with the lithium metal. The broader voltage hysteresis observed in the BP-2 system further highlights its inferior kinetic performance compared to CBP-2-Cu. The enhanced electrochemical performance of CBP-2-Cu can be attributed to its optimized polymer architecture and Cu incorporation, which collectively improve Li-ion conductivity and mechanical robustness, effectively suppressing dendrite formation. The incorporation of Cu­(TFSI)_2_ in the BP matrix not only enhanced ionic conductivity and interfacial stability but also showed no evident detrimental effects during the cycling tests performed here. Nevertheless, we recognize that the long-term chemical stability of Cu^2+^ in contact with the lithium metal is an important consideration, and future studies will further evaluate this aspect. These results confirm that CBP-2-Cu provides superior cycling stability and rate capability, making it a promising candidate for high-performance LMBs.

### Electrochemical Performance of CPEs with a Model Organic Cathode

A solid-state composite polymer electrolyte (CPE) presents an effective solution to the solubility issue of organic cathodes by physically restricting active material dissolution, thereby enhancing the cycling stability. In this study, we aim to demonstrate this concept using the CBP-2-Cu electrolyte. To further evaluate its adaptability, CBP-2-Cu was tested with an organic PTCDA cathode in a Li|CBP-2-Cu|PTCDA cell to assess its rate capability and compatibility. The PTCDA organic cathode exhibits two symmetric voltage plateaus during cycling, arising from its characteristic two-step, two-electron redox reaction. In the first step, one pair of carbonyl groups undergoes lithiation, followed by reduction of the remaining carbonyls to a fully lithiated state. The sharp and reversible plateaus reflect the structural stability of the perylene framework and its suitability for probing our CBP-2-Cu electrolyte in LMB systems (Figure S13). The discharge capacities exhibited a minimal decrease with the increasing current density for the Li|CBP-2-Cu|PTCDA delivering specific capacities of 128.1, 124.0, 122.2, and 121.3 mA h g^–1^ at 0.1, 0.2, 0.5, and 1 C-rates (Figure 6). The voltage profiles further confirm that CBP-2-Cu enables stable charge–discharge characteristics even at high C-rates, minimizing polarization and maintaining well-defined plateau regions (Figure S12). In contrast, the Li|liquid electrolyte (LE)|PTCDA cell exhibited significantly lower specific capacities of 109.5, 103.5, 97.3, and 95.8 mA h g^–1^ under the same conditions. For small organic molecule electrodes such as PTCDA, an initial decline in capacity is commonly observed due to dissolution, which is a major contributor to performance degradation. Notably, the CBP-2-Cu system outperformed the conventional liquid electrolyte counterpart, exhibiting significantly enhanced cycling stability and effectively suppressing dissolution-induced capacity loss. In contrast, the organic electrode with the conventional LE exhibited rapid capacity fading, with a pronounced decline occurring within the first 30 cycles. These results highlight the exceptional mechanical strength, ionic conductivity, and interfacial stability imparted by Cu incorporation in CBP-2-Cu, making it a promising electrolyte for next-generation LMBs. Its ability to enhance the cycling performance of organic cathodes further underscores its broad applicability and potential to enable high-performance, long-lifespan organic-based energy storage systems.

**Figure 6:**
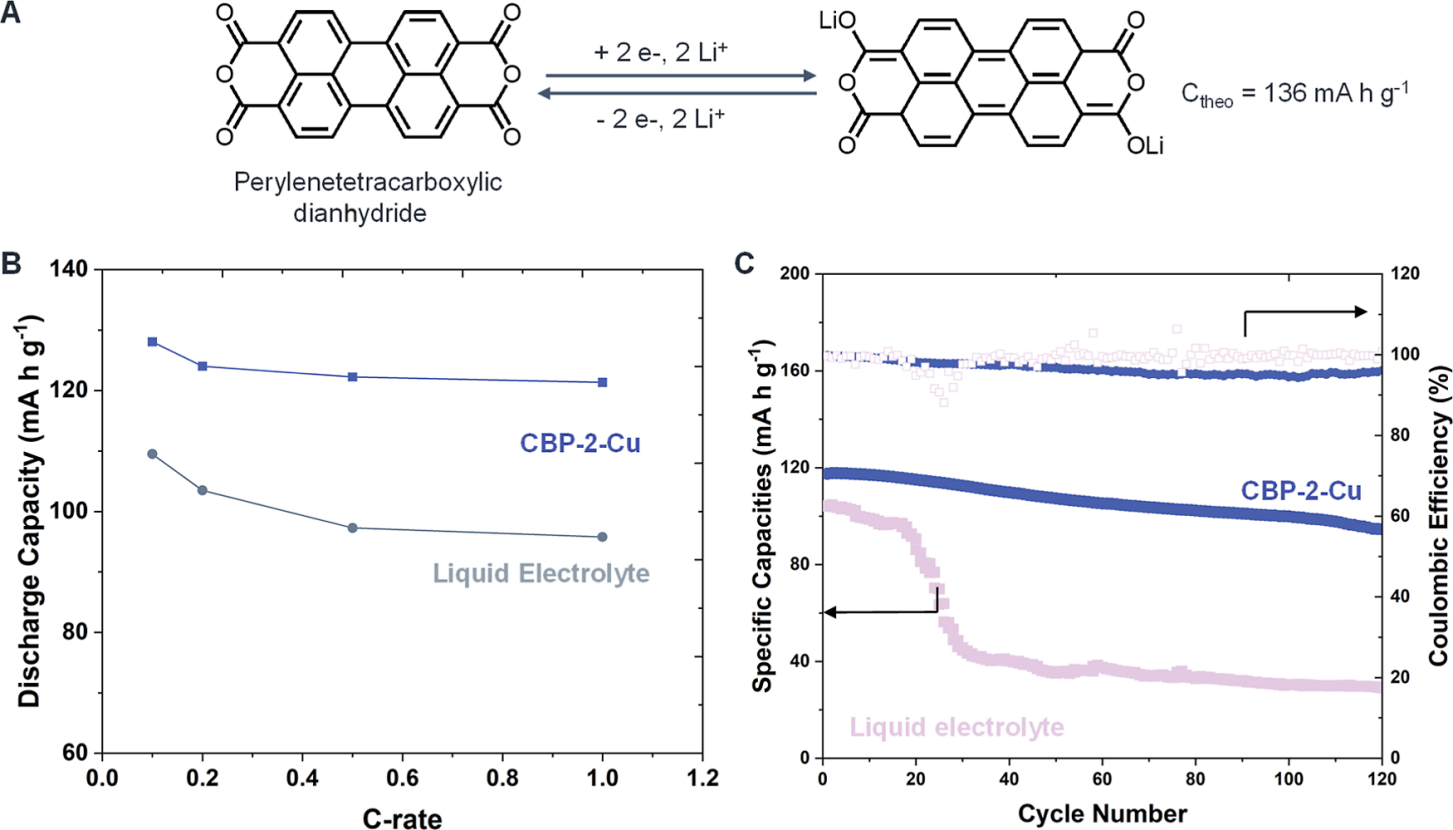
Electrochemical performance of CBP-2-Cu with an organic PTCDA cathode at room temperature. (A) Redox reaction of PTCDA, (B) discharge capacities at different c-rates, and (C) cycling stability at 0.5C rate.

## Conclusion

In this work, we successfully developed a CPE utilizing polyoxanorbornene-based BPs with PEO side chains enhanced by the incorporation of Cu­(TFSI)_2_. The bottlebrush architecture provided precise molecular control, enhancing mechanical robustness, while preserving the amorphous character necessary for efficient lithium-ion conduction. The addition of Cu­(TFSI)_2_ further disrupted the PEO crystallinity and modified the lithium coordination environment, resulting in a significant increase in the ionic conductivity. Electrochemical analysis demonstrated that the optimized CBP-2-Cu electrolyte maintained stable lithium metal cycling for over 500 h with low overpotentials, confirming its ability to effectively suppress dendrite growth. Full-cell evaluations using both inorganic (LiFePO_4_) and organic (PTCDA) cathodes showcased the broad applicability and superior electrochemical performance of CBP-2-Cu. Compared to conventional polymer electrolytes, the Cu-modified bottlebrush electrolyte demonstrated enhanced rate capability, higher reversible capacities, and improved cycling stability, even under challenging low-rate conditions, where dendrite formation is typically more pronounced. Furthermore, the electrolyte’s strong interfacial stability successfully mitigated the dissolution of organic cathodes, highlighting its potential to enable sustainable organic-based battery chemistries. Overall, this study underscores the importance of the molecular-level polymer design and targeted ion coordination strategies in the development of next-generation SPE. By combining mechanical robustness, high ionic conductivity, and versatile electrode compatibility, CBP-2-Cu represents a highly promising candidate for future LMBs, offering a viable path toward safer, higher-energy, and more durable energy storage systems.

## Supplementary Material



## Notes

Experimental details, materials, and methods; synthesis and characterization of PEO-based macromonomers and BPs; 1H NMR and FT-IR spectra; thermal analysis (TGA, DSC); rheological measurements; ionic conductivity data; LSV results; SEM images of cycled Li anodes; and electrochemical performance of Cu-enhanced CPEs (PDF)
