# Supplementary material for: Cu-Enhanced Bottlebrush Composite Polymer Electrolytes for Superior Mechanical and Electrochemical Performance
Source: ACS Appl Energy Mater. 2025 Oct 28;8(21):16040–50. doi: 10.1021/acsaem.5c02545 (PMC12606550; doi:10.1021/acsaem.5c02545)
Supplement: Supplementary file 1 [file ae5c02545_si_001.pdf]

Supporting Information

Cu-Enhanced Bottlebrush Composite Polymer Electrolytes for Superior Mechanical and Electrochemical Performance

So Young An[a], Brian Hu[a], Young-Geun Lee[b], Yuqi Zhao[b], Ting-Chih Lin[a], Jay. F. Whitacre[b,c]* and Krzysztof Matyjaszewski[a]*

[a] Department of Chemistry, Carnegie Mellon University, 4400 Fifth Avenue, Pittsburgh, Pennsylvania, 15213, United States
[b] Department of Materials Science and Engineering, Carnegie Mellon University, 5000 Forbes Avenue, Pittsburgh, Pennsylvania, 15213, United States
[c] Scott Institute for Energy Innovation, Carnegie Mellon University, 5000 Forbes Avenue, Pittsburgh, Pennsylvania, 15213, United States

* Correspondence:
whitacre@andrew.cmu.edu (J.F.W.), matyjaszewski@cmu.edu (K.M.)

Additional experimental details, materials, and methods; synthesis and characterization of PEO-based macromonomers and bottlebrush polymers; ^{1}H-NMR and FT-IR spectra; thermal analysis (TGA, DSC); rheological measurements; ionic conductivity data; linear sweep voltammetry (LSV) results; SEM images of cycled Li anodes; and electrochemical performance of Cu-enhanced composite polymer electrolytes.

Experimental procedures

Materials and Instrumentation:

All reagents were purchased from Sigma-Aldrich and used as received. ^{1}H-NMR and ^{13}C-NMR spectra were recorded using a Bruker Advance III 500 MHz. Chemical shifts are reported in ppm at room temperature using the solvent peaks of $CDCl_3$ at 7.26 ppm and DMSO-d_6 at 2.50 ppm. Gel permeation chromatography (GPC) measurements of polymers were performed using PSS columns with DMF as eluent at 50 °C and the flow rate of 1 mL min^{-1}. Linear poly(methyl methacrylate) standards were used for calibration. Thermogravimetric Analysis (TGA) was performed on TA instruments 2950 under a nitrogen atmosphere with a flow rate of 60 mL min^{-1}. The polymers' glass transition temperatures (T_g) were measured by Differential Scanning Calorimetry (DSC) with TA Instrument QA-2000 with a heating/cooling rate of 10 °C/min. Scanning Electron Microscope (SEM) was performed for cathode surface morphology investigation and conducted on a Quanta 600 FEG instrument. Images were acquired through an ETD detector at 30 kV with a spot size 3.5. Electrochemical measurements were performed using a Bio-Logic 16-channel VMP-3 multi-channel potentiostat/electrochemical impedance spectrometer or a Landt CT3001A battery testing system at room temperature. Electrochemical characterization was carried out using CR2032-type coin cells. All electrochemical impedance spectroscopy analyses were performed at a range of temperatures from 25 °C to 60 °C on a Bio-Logic 16-channel VMP-3 multi-channel potentiostat. Rheological measurements were performed on TA instruments (TA Instruments; New Castle, DE) DHR-2 stress-controlled rheometer. A 20 mm cone and plate configuration was used. To measure the viscoelasticity (G', G'') of the samples, the frequency sweeps (0.1 to 300 rad/s) were conducted under the constant value of strain amplitude 10% and 25°C. The 10% strain was confirmed to be in the linear viscoelastic region.

Synthesis of PEO-containing macromonomers. The synthesis of the macromonomer was based on a previously reported procedure with minor modifications. The oxanorbornene precursor (2.0 g, 12 mmol), mPEO-OH, M_n = 2000 g/mol (7.1 g, 7.1 mmol), and triphenylphosphine (2.2 g, 8.6 mmol) were added to a flask. The flask was backfilled with nitrogen, and anhydrous tetrahydrofuran (60 mL) was added by a syringe. The resulting solution was stirred and cooled in an ice-water bath. Diiospropyl azodicarboxylate (DIAD, 1.4 mL, 8.6 mmol) was added dropwise to the above solution using an additional funnel. The solution was brought to room temperature slowly and stirred overnight. The solvent was evaporated under reduced pressure, and the residue was dissolved in water. The aqueous solution was washed with ethyl acetate to remove unreacted monomer, mPEO-OH, reduced DIAD, and triphenylphosphine oxide. Water was then removed on a lyophilizer to give a white solid. Yield: 84%, ^{1}H-NMR (500 MHz, $CDCl_3$)

Synthesis of BPs. Under an inert gas atmosphere, Grubbs third generation catalyst (G3) was dissolved in a minimal amount of freeze-pump-thawed dichloromethane and added quickly to a solution of the PEO-containing macromonomer in dichloromethane. The resulting solution was stirred for 2 hours or 18 hours depending on the completion of polymerization. Excess ethyl vinyl ether was added to the flask, stirring the solution for 2 hours. The solvent was removed under a vacuum to afford a faint brown solid. Purification was performed by repeated precipitation in hexane/ethyl ether = 1:1 (v/v), afforded a faint yellow solid or off-white solid. Yield: 90-93%. ^{1}H-NMR of P2 (400 MHz, $CDCl_3$)

Preparation of composite polymer electrolytes. LiTFSI and BPs were mixed in a certain ratio in anhydrous tetrahydrofuran and stirred for 1 hour. Typically, the average molar ratio of ethylene oxide units (EO) to lithium-ions was varied from 10/1 to 20/1. Lastly, $Cu(TFSI)_2$ (wt % varying from 0% to 5 %) to the mixture. Then the mixed solution was drop-cast onto a substrate (stainless

steel or lithium metal) in the glovebox (level of H_2O and $O_2 < 1$ ppm). A Teflon ring with a thickness of 0.8 mm was used so that the thickness of the electrolyte remains relatively constant between the samples. The THF was evaporated on a hot plate with a temperature of 40 °C for 1 hour. Afterward, the samples were placed into the glovebox's vacuum chamber and dried under vacuum for three hours. Note that the total amount of THF used per sample (or battery) is less than 0.5 mL.

Ionic conductivity measurements. The ionic conductivity measurements of the polymer electrolytes were carried out using the AC impedance spectroscopic technique. The polymer electrolytes were sandwiched as Stainless steel/composite polymer electrolytes (CPE)/Stainless steel in a glovebox with an argon atmosphere (level of H_2O and $O_2 < 1$ ppm). The measurements were performed at temperatures ranging from 25 to 60 °C. The cell was thermally equilibrated for 30 min at each temperature point before measurements. The AC impedance spectra were recorded over the 0.01 to 10^6 Hz frequency range with a voltage amplitude of 100 mV. The ionic conductivity values were derived from the measured resistance using **Equation S1.**

Linear Sweep Voltammetry (LSV) curves. The electrochemical window stability of the polymer electrolyte was evaluated using Li/CPE/stainless steel (asymmetric battery configuration) by sweeping the voltage from 3.0 to 5.5 V with a scan rate of 0.1 mV/s.

Electrode preparation and coin cell assembly. Electrochemical characterization was carried out using 2032-type coin cell. LFP active material (or organic cathode, PTCDA) was combined with carbon Super P and PVDF in an 80:10:10 (w/w/w) ratio and suspended in NMP at 0.05 g/mL concentration. The slurry was sonicated for 30 minutes, stirring every 10 minutes to homogenize. The slurry was then loaded into a spray gun (Master, Airbrush, G22). With a constant airflow, the

liquid was sprayed uniformly on an aluminum foil preheated to 150 ºC. The as-obtained coated cathode film was taken off from the heating plate and dried at 80 ºC in a vacuum oven overnight. An electrode punch was used to cut the electrodes to a 10 mm diameter (0.9 mg cm^{-2} active material loading on the current collector). Lithium foil with a diameter of 11 mm was used as the anode. The coin cells were assembled using P1 or P2 polymer electrolyte, a stainless spacer (0.5 mm thickness), and one spring for optimal cell compression. The cells were hermetically sealed using a pressure-controlled electric crimper (MTI Corporation). For Li/SPE/Li cell configuration, SPE P1 or P2 was sandwiched between two 11 mm diameters of lithium electrodes. The electrolyte was allowed to have good contact with the electrodes for 10 hours before cycling all batteries. Prior to the cycling experiment, the battery was subjected to three formation cycles at 0.1 C-rate from 2.5 V to 3.8 V. Rate test and long-term cycling testing of Li|SPE|LFP cell were conducted on BioLogic channels at room temperature. For post-mortem analysis of cycled cells, coin cells were disassembled in a glovebox, then the lithium electrode with polymer electrolyte was cut into halves using a blade. The electrodes with the polymer electrolyte layer were directly used for SEM analysis.

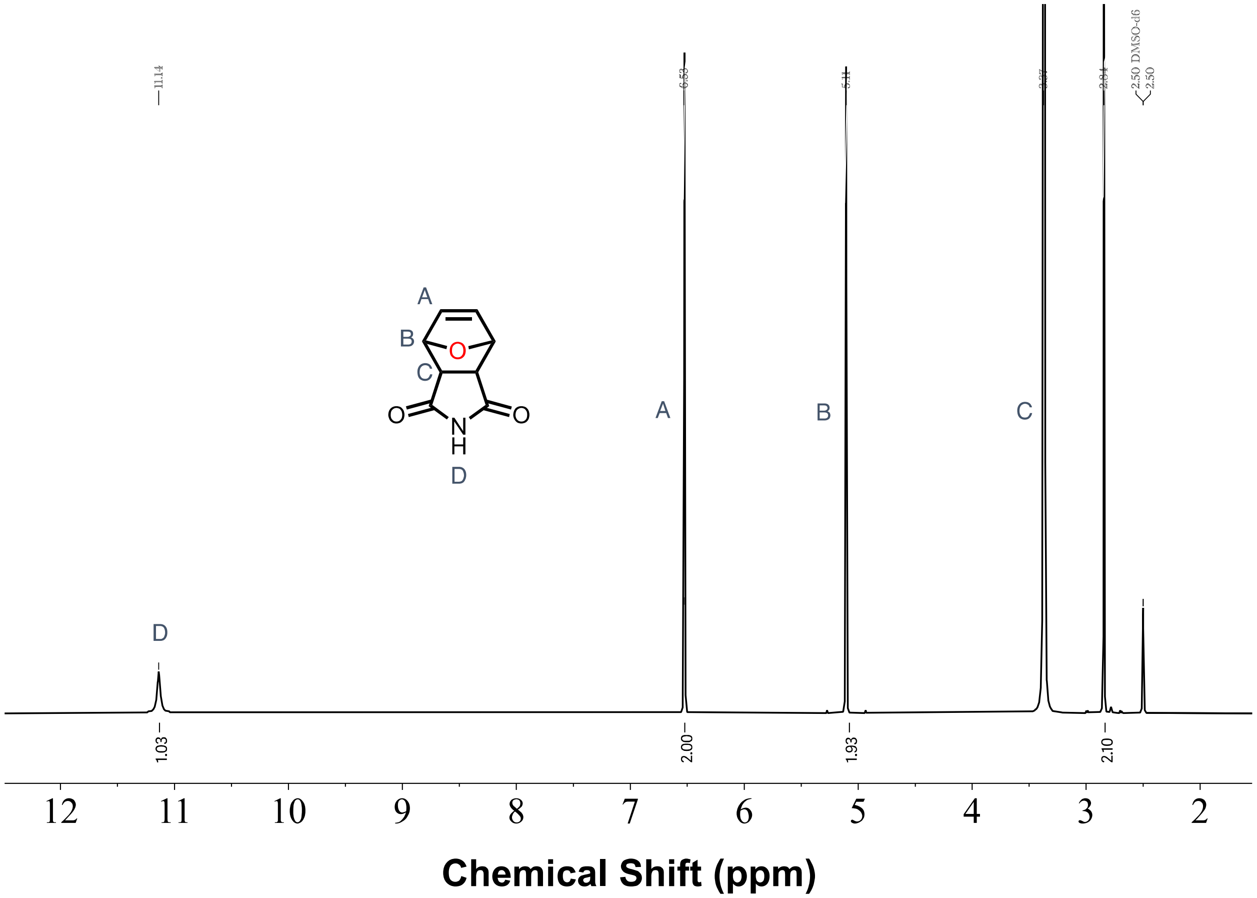

Figure S1. ^{1}H-NMR spectrum of oxanorbornene precursor.

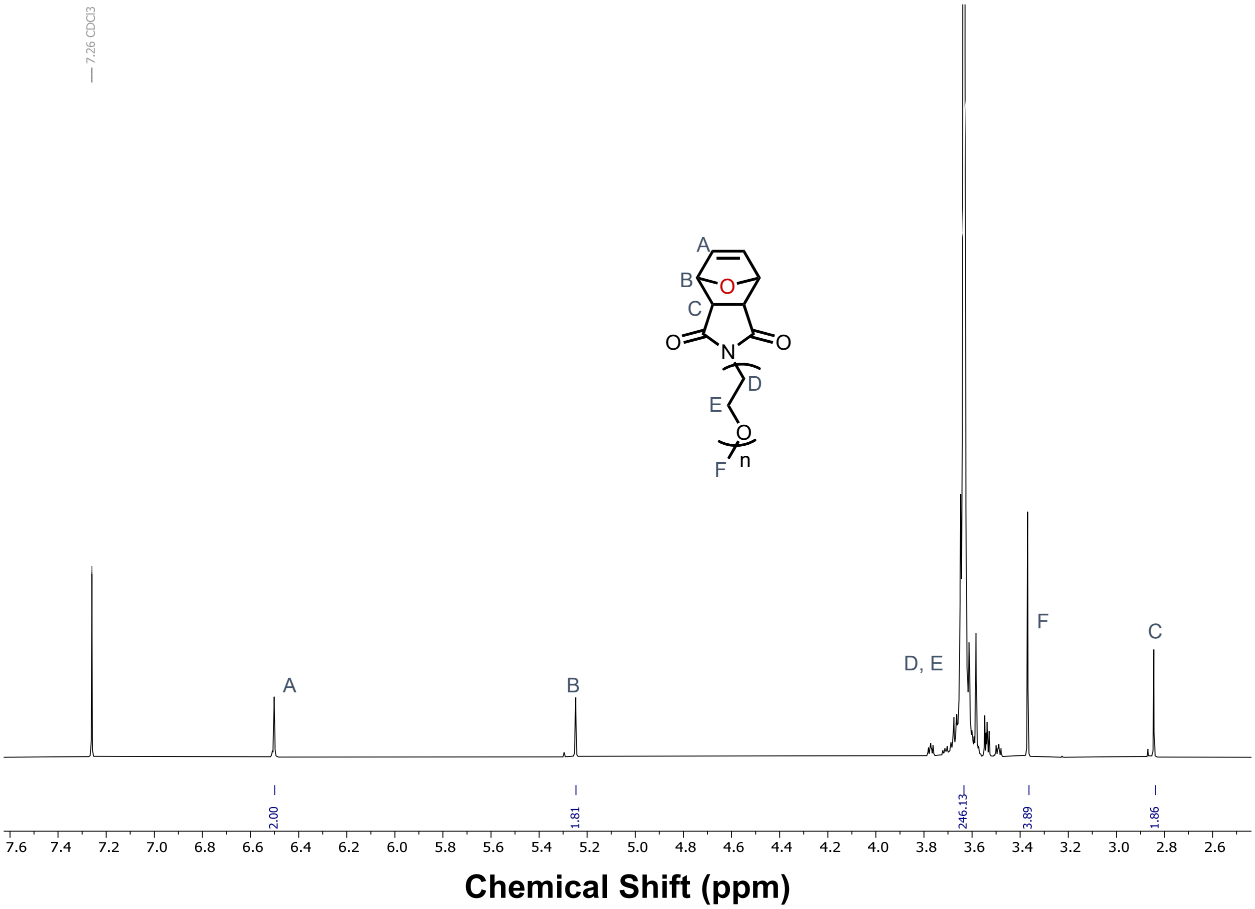

Figure S2. ^{1}H-NMR spectrum of a PEO containing macromonomer (M_n ~2147 g/mol) for a BP.

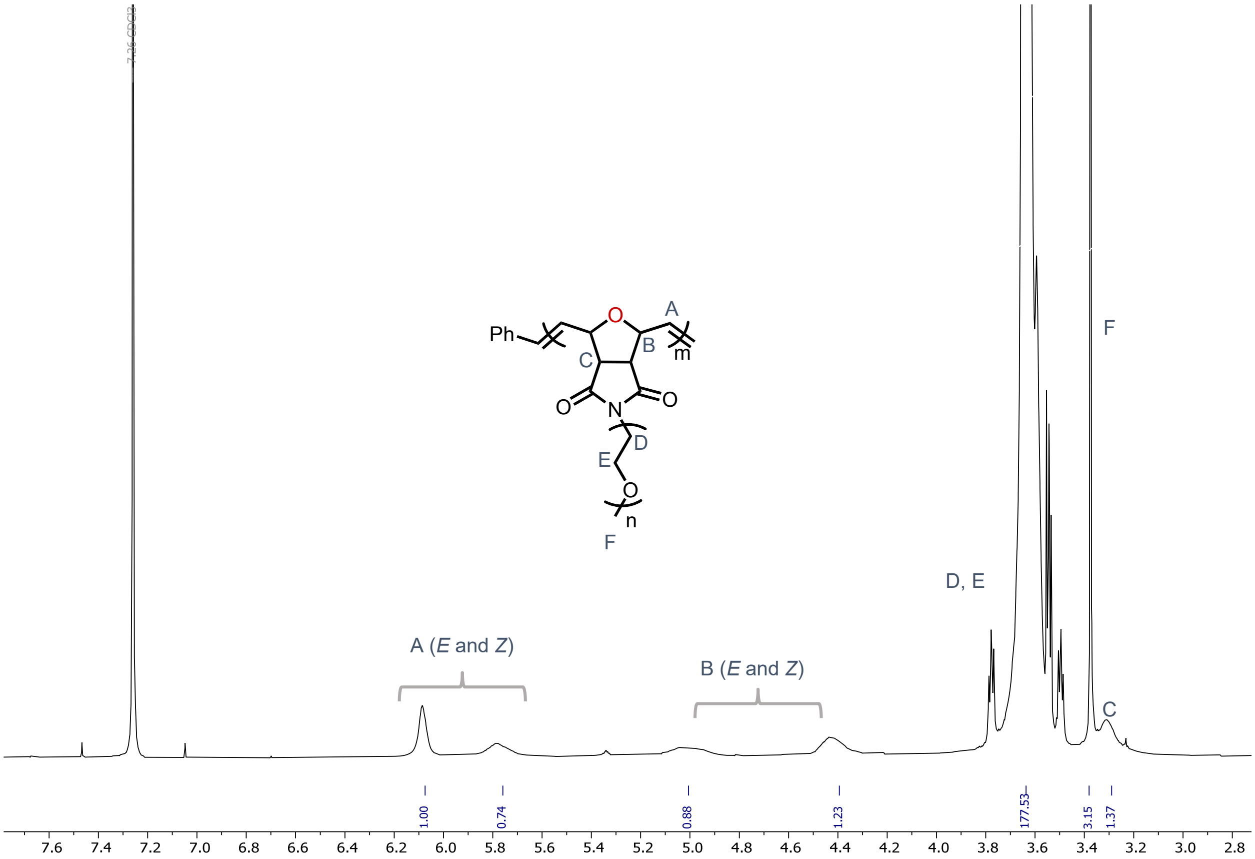

Figure S3. Representative ^{1}H-NMR spectrum of BP1.

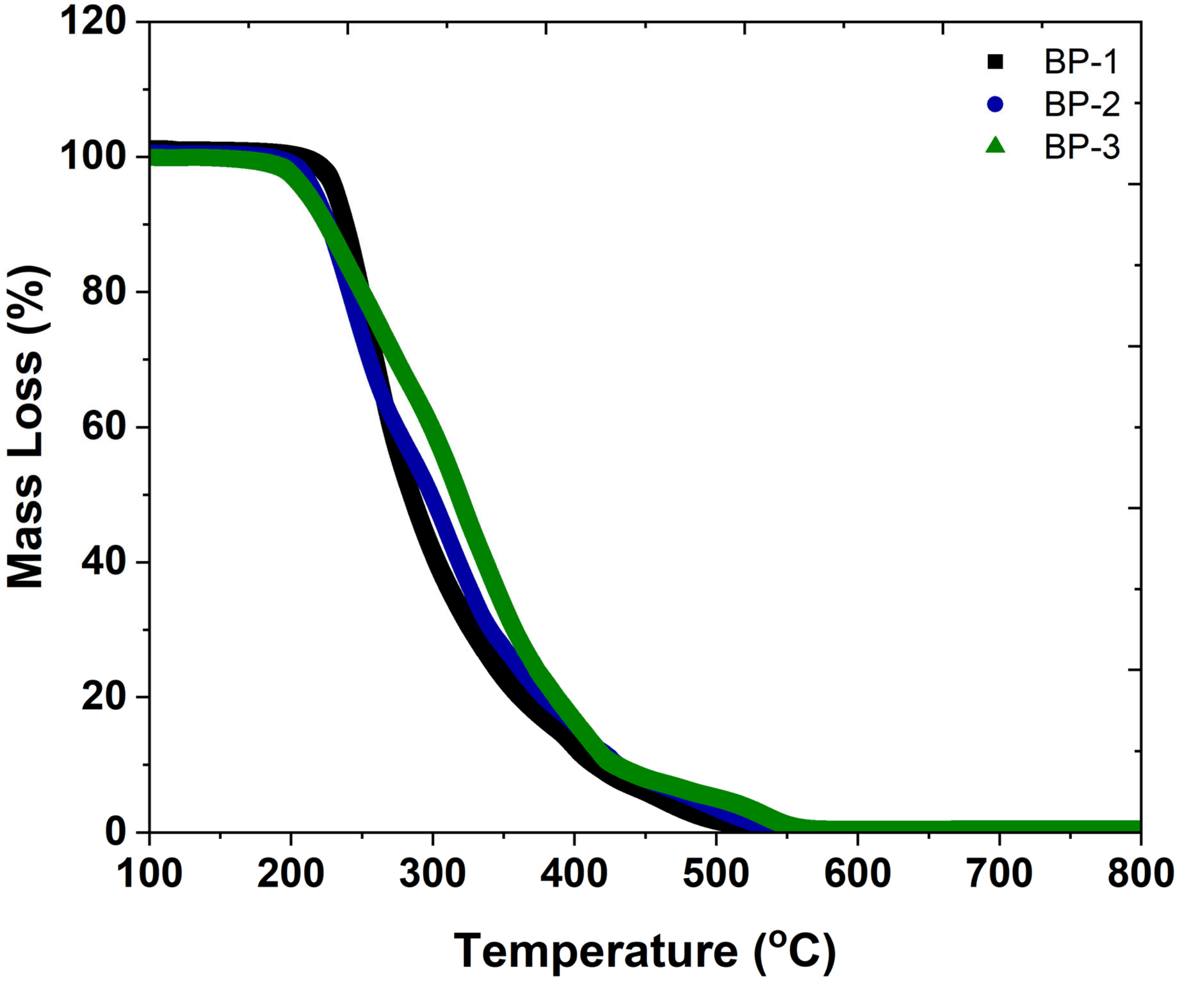

Figure S4. Thermal properties of BP-1, BP-2 and BP-3.

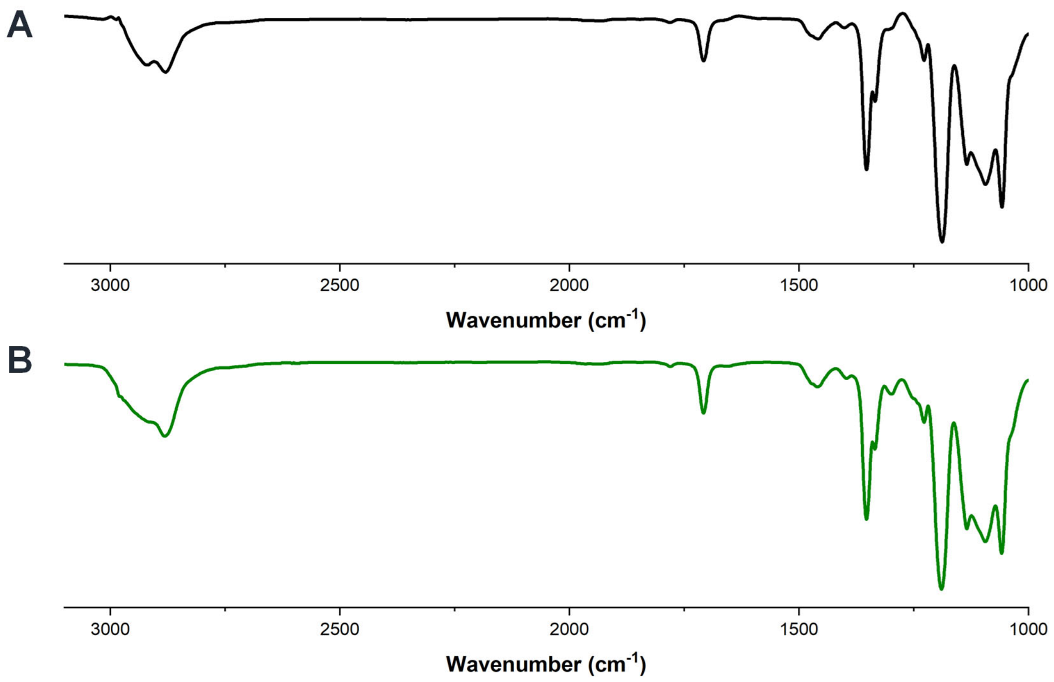

Figure S5. FT-IR trace of (A) CBP-2 without CuTFSI and (B) CBP-2 with 2 wt % CuTFSI

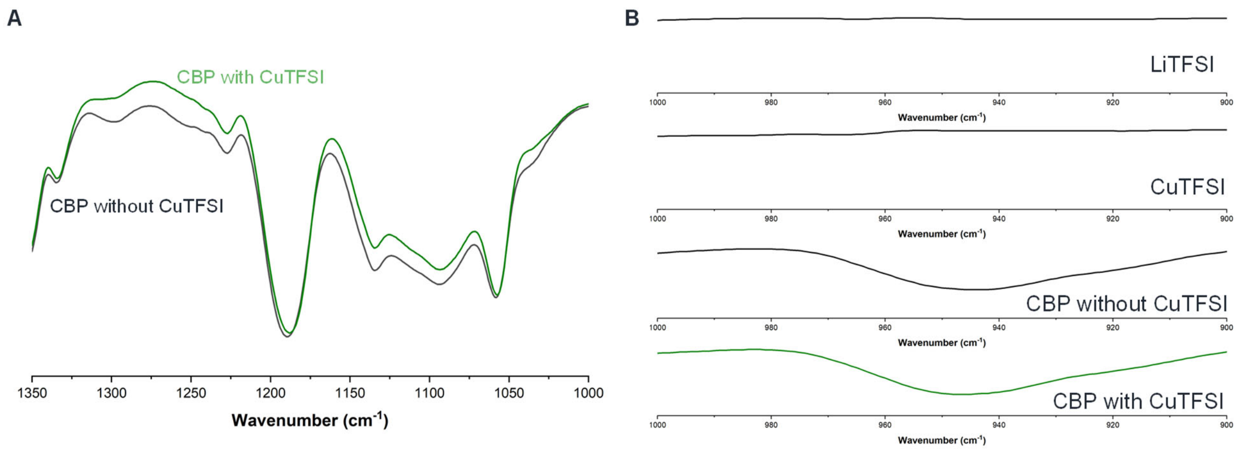

Figure S6. Expanded view of the FT-IR spectrum CPB-2-Cu (with 2 wt% $Cu(TFSI)_2$) and BP-2 (without CuTFISI) in the selected wavenumber region.

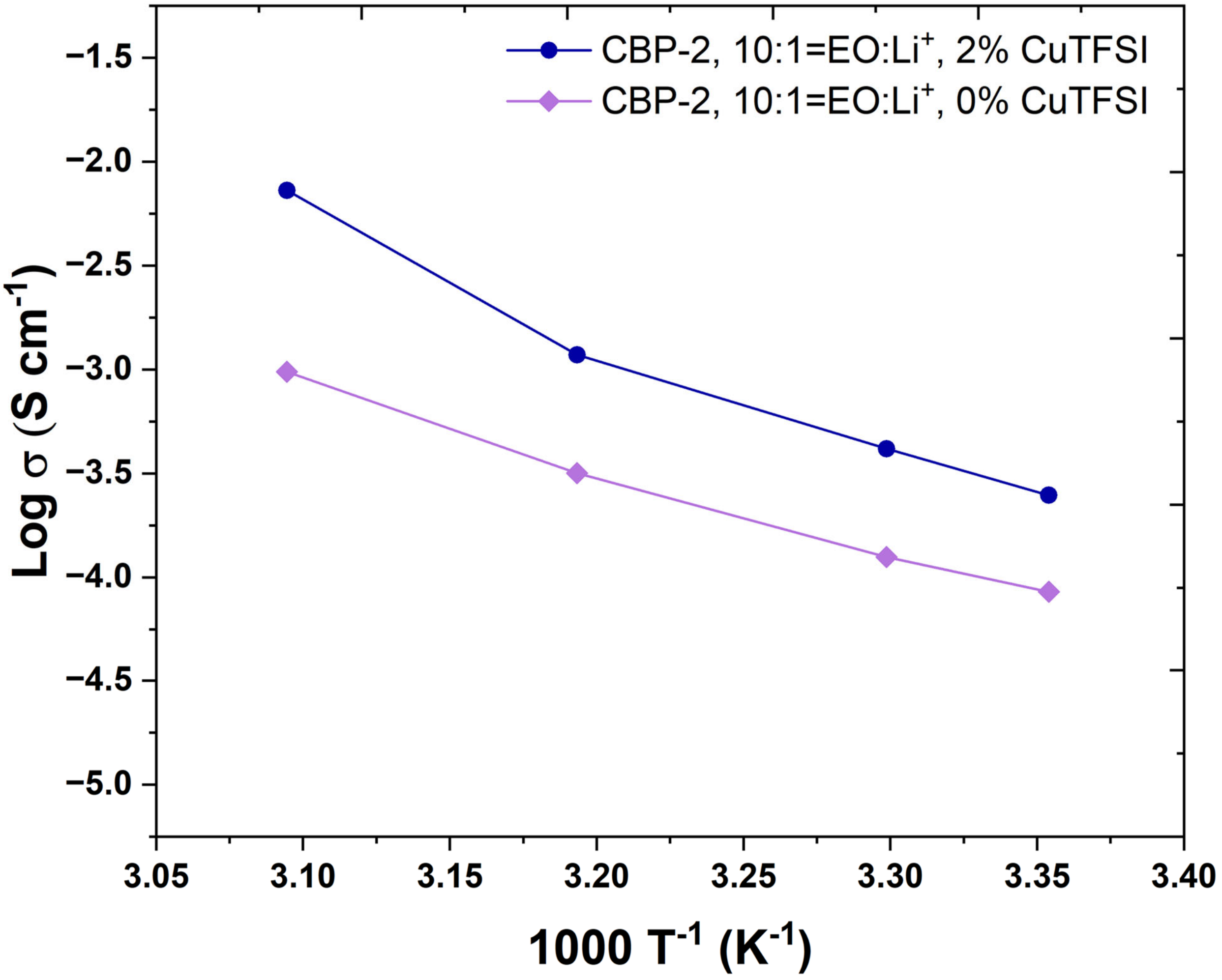

Figure S7. Ionic conductivity of composite polymer electrolyte CPB-2-Cu (with 2 wt% $Cu(TFSI)_2$) and BP-2 with and without $Cu(TFSI)_2$.

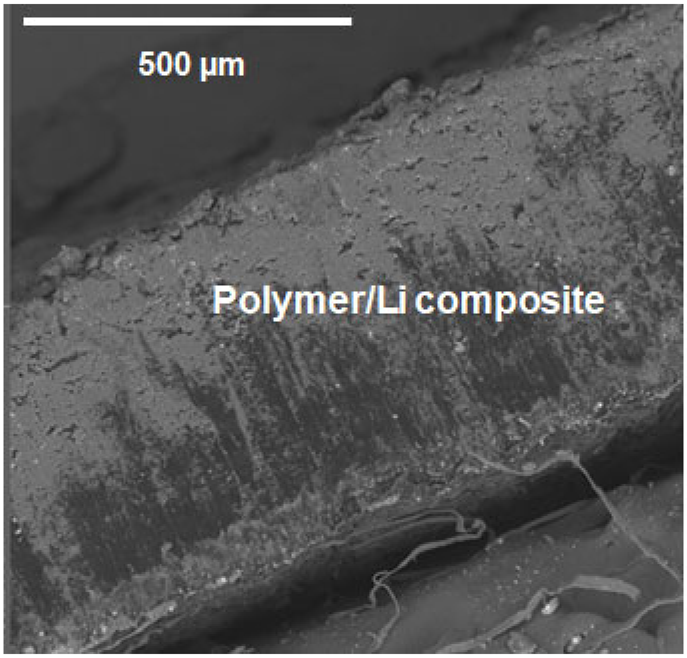

Figure S8. SEM images of cycled lithium anode from Li|BP-2|Li after 100hr

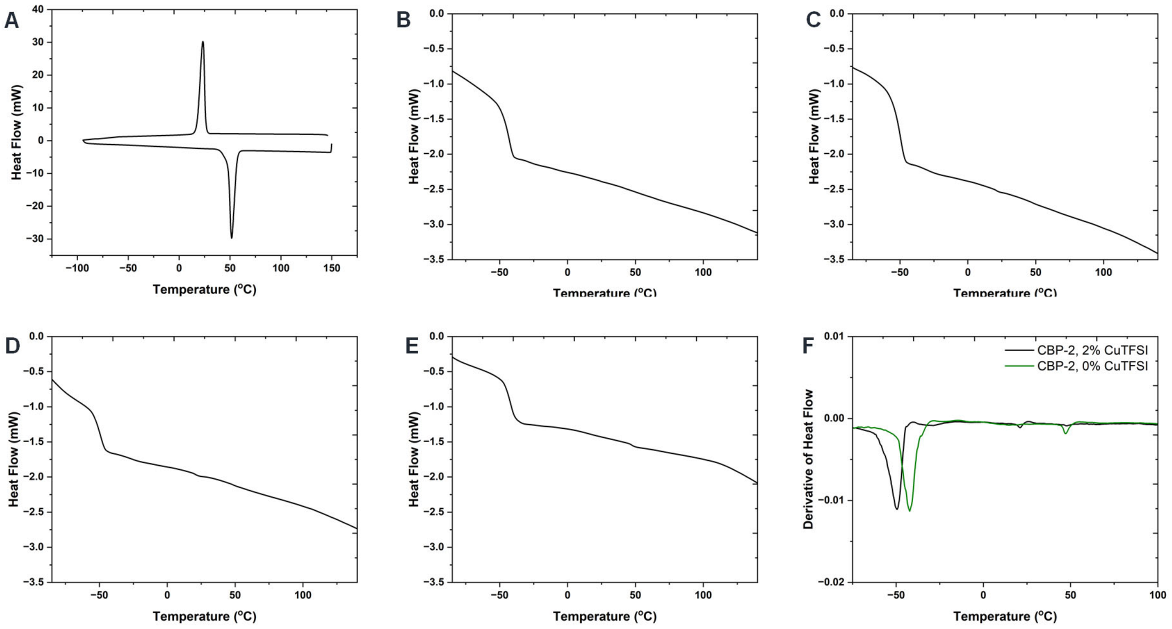

Figure S9. Differential scanning calorimetry (A) BP-2 (B) CBP-2 with 1 wt% of $Cu(TFSI)_2$ (C) CBP-2 with 2 wt% of Cu(TFSI)2 (D) CBP-2 with 5 wt% of $Cu(TFSI)_2$ (E) CBP-2 with 0 wt% of $Cu(TFSI)_2$ (F) comparison of derivatives of heat flow for CBP-2 with and without $Cu(TFSI)_2$

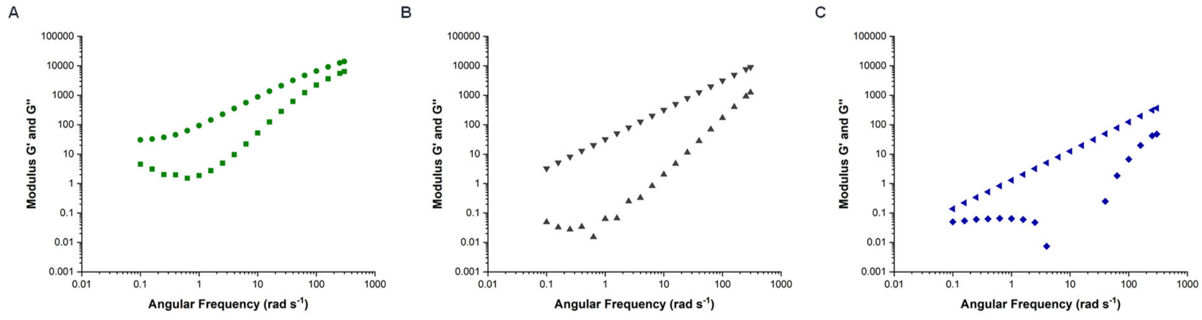

Figure S10. Frequency sweep test under the constant value of strain amplitude 10% at 25 °C Storage modulus and loss modulus as a function of angular frequency for (A) CBP-2 with 2 wt % $Cu(TFSI)_2$ (B) CBP-2 without $Cu(TFSI)_2$ and (C) PEO SPE

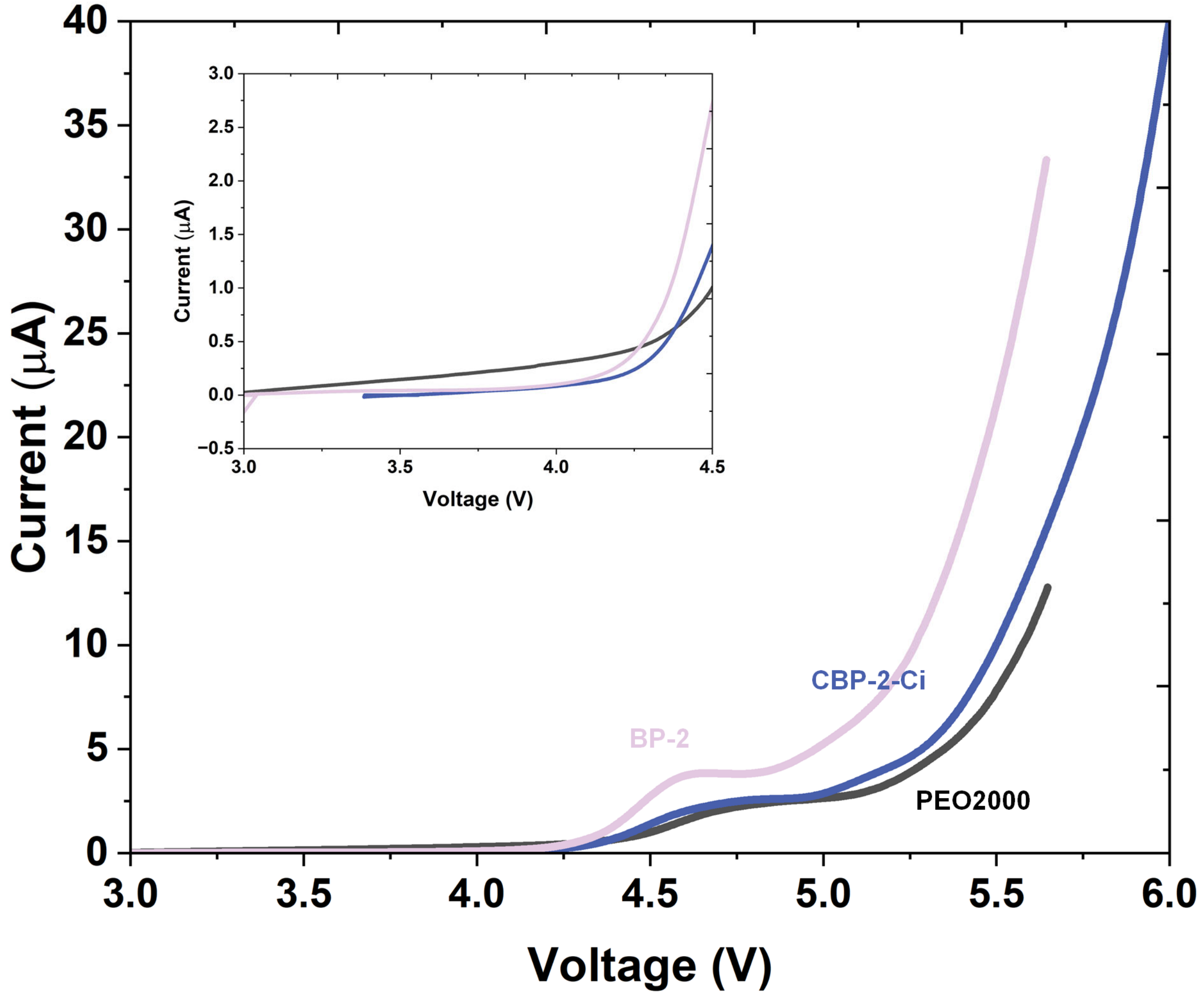

Figure S11. LSV curve of composite polymer electrolytes.

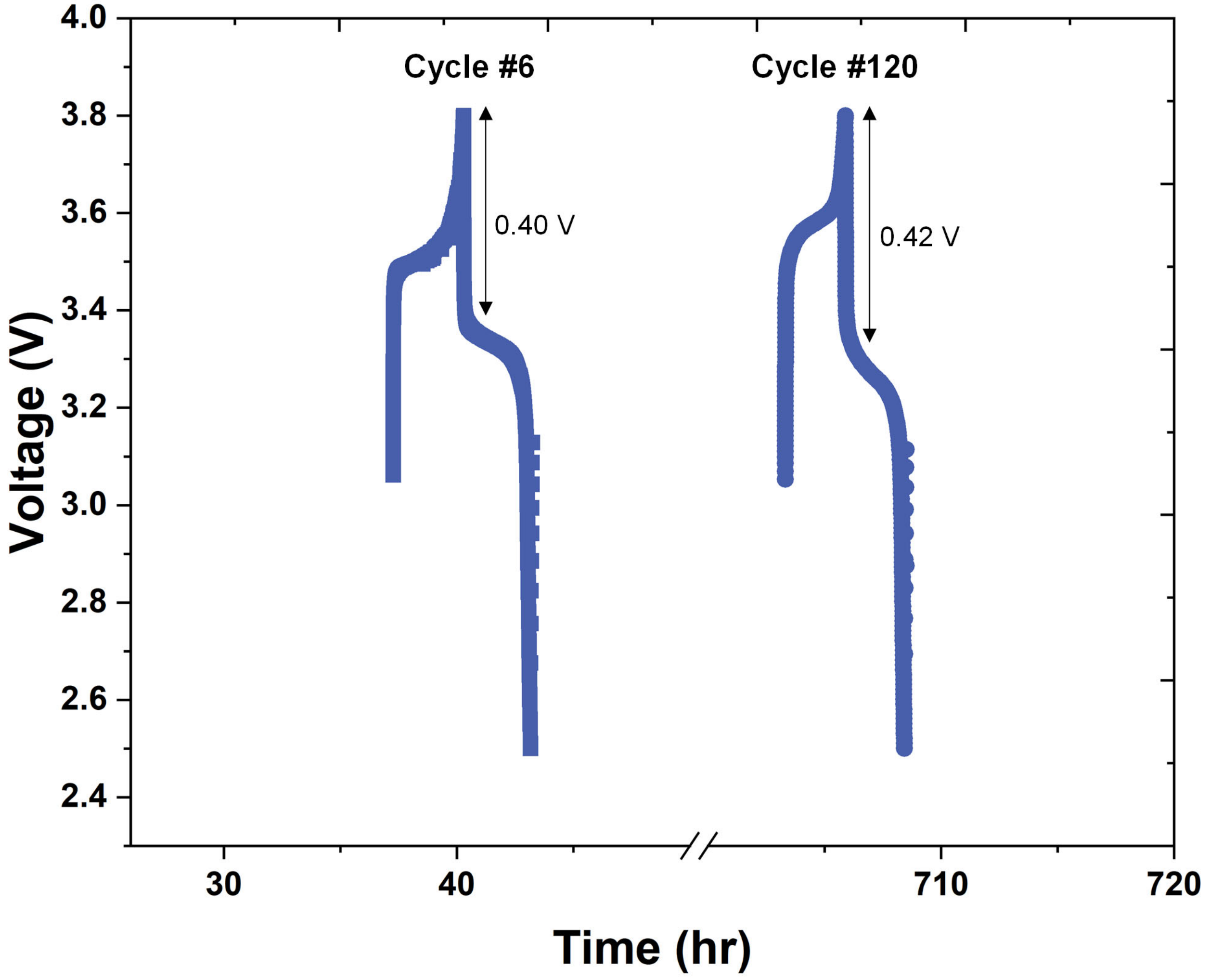

Figure S12. Voltage profiles during long-term cycling at 0.5C for CBP-2-Cu at different cycle numbers.

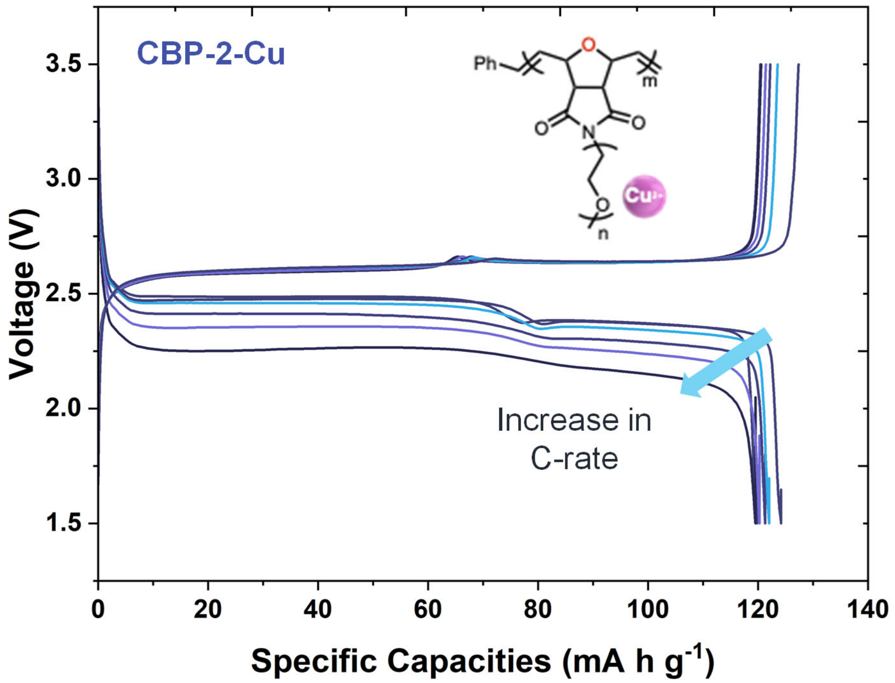

Figure S13. Charge discharge voltage profiles for the Li|CBP-2-Cu|PTCDA with increase in C-rates.

$$\sigma = \frac{L}{R \times S} \quad \text{(Equation S1)}$$

Where *L* is the thickness of polymer electrolytes, *A* is the area of the electrolyte, and *R* is the impedance determined from the Nyquist plot.
